# RhizoNet segments plant roots to assess biomass and growth for enabling self-driving labs

**DOI:** 10.1038/s41598-024-63497-8

**Published:** 2024-06-05

**Authors:** Zineb Sordo, Peter Andeer, James Sethian, Trent Northen, Daniela Ushizima

**Affiliations:** 1 Computing Sciences Area, Lawrence Berkeley National Laboratory, Berkeley, CA 94760 USA; 2 Biosciences Area, Lawrence Berkeley National Laboratory, Berkeley, CA 94760 USA; 3 Department of Mathematics, University of California, Berkeley, CA 94720 USA; 4 Berkeley Institute for Data Science, University of California, Berkeley, CA 94760 USA; 5 Bakar Institute, University of California, San Francisco, CA 94143 USA

**Keywords:** Biotechnology, Computational biology and bioinformatics, Plant sciences, Environmental sciences, Energy science and technology

## Abstract

Flatbed scanners are commonly used for root analysis, but typical manual segmentation methods are time-consuming and prone to errors, especially in large-scale, multi-plant studies. Furthermore, the complex nature of root structures combined with noisy backgrounds in images complicates automated analysis. Addressing these challenges, this article introduces RhizoNet, a deep learning-based workflow to semantically segment plant root scans. Utilizing a sophisticated Residual U-Net architecture, RhizoNet enhances prediction accuracy and employs a convex hull operation for delineation of the primary root component. Its main objective is to accurately segment root biomass and monitor its growth over time. RhizoNet processes color scans of plants grown in a hydroponic system known as EcoFAB, subjected to specific nutritional treatments. The root detection model using RhizoNet demonstrates strong generalization in the validation tests of all experiments despite variable treatments. The main contributions are the standardization of root segmentation and phenotyping, systematic and accelerated analysis of thousands of images, significantly aiding in the precise assessment of root growth dynamics under varying plant conditions, and offering a path toward self-driving labs.

## Introduction

Biofuels represent renewable energy sources extracted from organic materials, such as plants or plant remnants, and can serve as an eco-friendly substitute for conventional fossil fuels^1,2^. To improve the yield of bioenergy crops, studies have investigated plant traits^3–5^, as well as the role of special nutrients in the promotion of plant growth^6^. Root growth and architecture can be critical determinants of crop productivity and sustainability^7^. Gaining knowledge of plant roots can support research on optimizing nutrient accessibility, improved nutrient absorption, and enhanced plant growth and biomass yield.

Examining root morphology directly within soil is technically challenging due to soil opacity. Instead, roots are typically excavated and imaged using flatbed scanners. However, this approach excludes sequential imaging of the same root system at different points in time. To observe roots growth and their interactions in the soil, researchers have often relied on specialized instruments known as minirhizotrons^8^. With early versions dating from the 1970s^9,10^, current minirhizotrons consist of transparent tubes or chambers that are vertically inserted into the soil, enabling non-destructive monitoring and capturing of images of plant roots as they evolve in their natural environment. RhizoVision^10^ software has been successfully used for analyzing scans and photographs of excavated roots.

While minirhizotrons provide valuable insights, they possess limitations such as restricted depth observation and interference with nearby roots. Additionally, they require complex data analysis and are best suited for transparent soils. To overcome opacity, several systems have been developed, notably the GLO-ROOTS system, which uses advanced imaging and cheminluminescence^11^ to allow for the imaging of roots directly within soils.

**Figure 1:**
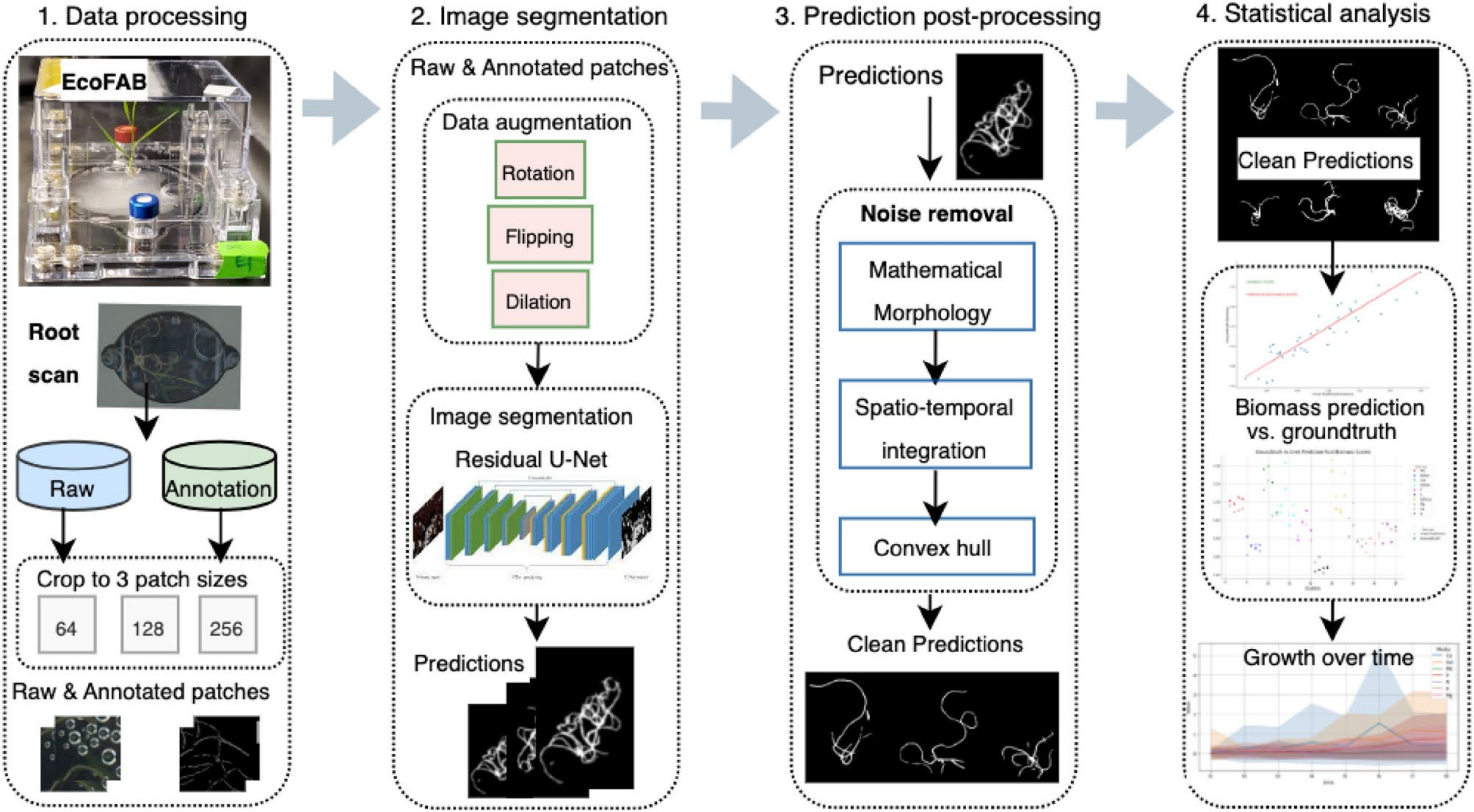
RhizoNet: proposed pipeline for root segmentation and root biomass estimation from time-resolved EcoFAB images.

Another system for plant monitoring is the EcoFAB (Ecosystem Fabrication)^12,13^ illustrated at the top left of the Fig. 1, a novel device that allows hydroponic cultivation and in-situ plant imaging. One of the pressing needs to optimize EcoFAB’s capabilities is to enable the detection of plant roots over time and quantification of their biomass using computer vision. However, some of the challenges in automating image segmentation include:

- *Fine-scale structures:* Roots display complex patterns in shades of green and brown, interwoven, and tangled.
- *Complex branching:* Roots exhibit varying calipers, can split and bifurcate, complicating segmentation.
- *Bubbles and condensation:* Water droplets and background moisture add different textures and visual artifacts.

## Imaging, machine learning, and RhizoNet

As one approach, threshold-based methods have been effective in numerous scenarios, as evidenced by recent studies^14–16^, however, they often rely on a significant contrast between the foreground and background. This contrast is typically achieved by methods such as staining or controlled lighting adjustments. Yet, such grayscale thresholding techniques fall short in the unique environment of EcoFABs. Challenges such as condensation and the interference of delicate leaves casting shadows significantly hinder the visibility of the roots.

More sophisticated approaches, such as semantic segmentation techniques, particularly those built on deep learning encoder-decoder architectures, have exhibited remarkable results in a variety of scientific fields^17,18^, and U-Net-based approaches^18–20^ have shown exceptional performance when applied to plant classification tasks^9,21, 22^. Despite their effectiveness in multiple vision-centric tasks, U-Nets sometimes struggle with the complex fine structures seen in high-resolution EcoFAB images. For example, our explorations with the RootPainter software^22^ yielded acceptable results for images with minimal artifacts, compounded by the need for a large number of annotations. However, when using complex root pictures, such as those coming from EcoFAB scans, we encountered several challenges in segmenting the roots, particularly when condensation was present around the region of interest.

Our goal in this paper is to develop a new approach to segmenting plant roots that is sensitive to thin structures. Here, we present “RhizoNet”, our proposed computational workflow (Fig. 1) designed to adapt a convolutional neural network (CNN) to a unique capability for studying plant roots. Demonstrating the versatility of semantic segmentation algorithms across different domains, we highlight their application in the analysis of plant roots grown in EcoFABs. Furthermore, the feasibility of incorporating RhizoNet into a self-driving plant monitoring system is examined by demonstrating its capabilities under demanding conditions.

RhizoNet was developed, in part, by exploiting our previous work, including semantic segmentation methodologies such as our IHCNet^19^ applied to immunohistopathology to detect and validate new radioactive tags for Alzheimer’s disease. Next, our batteryNet^23^ framework can analyze batteries to inspect multiphase materials, using microCT and time-lapsed 3D data, and has proven invaluable for identifying lithium agglomeration and evaluating electrolyte state during battery operation. Both RhizoNet and BatteryNet employ a MONAI-based Residual U-Net architecture, which is an architecture that has proven successful in our applications and has demonstrated robust performance across varying domains, including both root segmentation in agronomy and electrode material segmentation in battery research.

The incorporation of residual connections within the U-Net architecture addresses a critical challenge in deep neural networks: the problem of vanishing gradients^24^, which can impede the training of deep networks, especially when learning to identify and segment intricate structures like plant roots. These residual connections help in preserving the gradient flow through the network, thus allowing for deeper models that can learn more detailed and subtle features of root structures, such as fine branching patterns.

The RhizoNet approach involves a fully-automated analysis of time-resolved EcoFAB images by aiming at fine structures defined by a few pixels; this allows the algorithm to handle the labyrinthine and dynamic nature of root systems within EcoFABs. By refining both the pre-processing and post-processing stages, we significantly enhanced the accuracy of our segmentation outcomes.

Figure 1 illustrates the computational pipeline for processing roots of *Brachypodium distachyon* plants that are subjected to various conditions of nutrient deprivation for approximately 5 weeks. Root images are captured at different time points, at intervals of 3 days to a week, as illustrated in Supplementary Fig. S.2. Each plant that grows in its respective chamber is monitored for weeks, with images acquired using a high-resolution flatbed scanner that provides information on the evolution of the plant over time within an EcoFAB.

At its core, RhizoNet uses a Residual U-Net architecture combined with a convex hull to deliver root segmentation specifically adapted for EcoFAB conditions. Our approach bridges the gap between traditional root analysis methods and the unique demands of high-resolution images from controlled studies of nutrient deprivation with a hydroponic system, enabling more precise and informative insights into root growth and plant responses.

## Results

EcoFAB images present an assortment of information about the root system; however, fully automated root segmentation requires complex methods to manage the difficulties posed by physical phenomena such as bubbles, droplets, reflections, and shadows. These inherent issues are particularly pronounced at smaller spatial scales, where fine structures can be a pixel width, which makes even manual annotation a major hurdle for human-experts.

In theory, when using smaller image patches, each neuron in the early layers of the artificial neural network has a smaller receptive field (the size of the region in the input that produces the feature) compared to processing the entire image when the network is exposed to a more global context during its learning process. The former enriches the latent space with a diverse set of feature vectors, conveying fine details about the image and can therefore improve generalization on unseen EcoFAB images, as illustrated in Fig. 3, and Supplementary Fig. S.1. As we investigated different strategies to train RhizoNet, we observed an improvement in the evaluation metrics for smaller patch sizes, that is, small inputs increase the robustness of the model since it is able to focus on thin objects and capture fine patterns despite the various artifacts and subtle nuances.

**Table 1:** RhizoNet’s segmentation evaluation before image post-processing: each line indicates performance given a model instance created by training the network using a specific patch size from Exp 2.

Patch size	Accuracy	Precision	Recall	IOU
(64,64)	71.3 ± 0.58	72.3 ± 0.58	96.3 ± 1.53	70
(128,128)	57	56.3 ± 0.58	96 ± 0.9	55 ± 0.85
(256,256)	50.3 ± 1.53	45 ± 1.73	88 ± 1.0	43 ± 1.73

In practice, small patch sizes also prevent class imbalance because they benefit from our strategy to unbias class distributions, which removes sparsely labeled patches, that is, patches with less than 20% of annotated pixels. It is important to emphasize that the EcoFAB images often exhibit more than 80% of pixels belonging to background as opposed to less than 20% of pixels from roots. Although the same training approach is used for all models, its effect is more pronounced on smaller patches as it avoids regions largely filled with black background, thus fostering improved class balance. This reasoning supports the evidence illustrated in Tables 1 and 2, showing high accuracy, precision, recall and IOU for smaller patch sizes. Additional details about each example in Table 2 also appear in Supplementary Table S.1. Figure 3 also highlights the influence of patch size in the algorithm’s ability to distinguish roots from other objects or artifacts.

**Table 2:** Evaluation metrics (Accuracy, Precision, Recall, IOU and AUC) of 6 unseen images of Exp 2, predicted and processed by each RhizoNet model.

	Accuracy	Precision	Recall	IOU	AUC
Model patch size 64	99.25 ± 0.24	96.82 ± 3.24	34.18 ± 6.6	33.85 ± 6.55	98.1 ± 1.77
Model patch size 128	99.15 ± 0.37	94.78 ± 3.88	32.1 ± 7.27	31.48 ± 6.89	96.97 ± 2.01
Model patch size 256	98.65 ± 0.37	99.03 ± 2.08	21.93 ± 3.08	21.9 ± 3.15	98.67 ± 1.56

Here, metrics are evaluated on full-size images rather than patches, as in Table 1.

To assess the performance of our predictions, we juxtaposed the predicted root biomass against the actual measurements from the final acquisition date for each media type. A linear regression analysis was conducted between the predicted biomass and the manually measured root weight using a scale. This analysis resulted in a correlation of 71.29% and a coefficient of determination (R^2^) of 84.43%. It is important to note that certain outliers remained, likely due to the presence of artifacts around and over the roots, which can mimic roots in both size and color, thus impacting the results.

**Figure 2:**
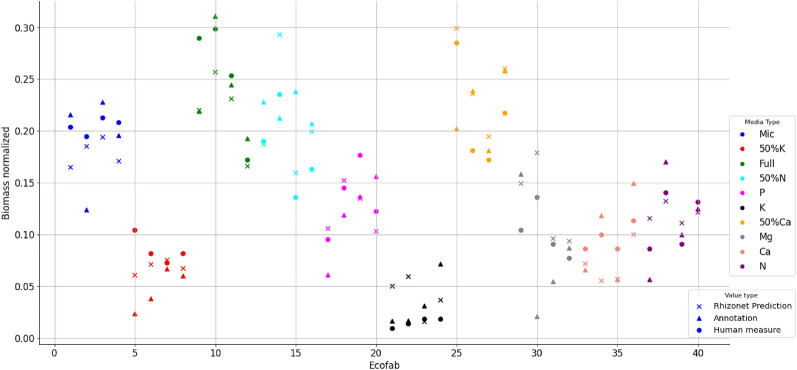
Root biomass: comparison between root weight (measured by a human with a scale), root area calculated from images (annotated manually by humans) and RhizoNet predictions; the colors indicate different media type for each plant in Exp 2; different symbols indicate different methods for the calculation of root biomass, shown in the legend under “value type”.

Regarding the comparison of root weight measurements taken manually with a scale and those derived from automated segmentation, we noted a significant correlation of 91.04% and a coefficient of determination of 82.87%. This improvement of automated segmentation over manual annotations, as highlighted in Fig. 2, illustrates the difficulty faced by human annotators in distinguishing root pixels from noisy artifacts. This challenge is particularly pronounced given the similar slender structures and colors of these elements.

In addition, the Residual Mean Squared Error (RMSE) was calculated to capture systematic errors within the model. In the case of scaled root weight measurements and the annotated biomass, we obtained a RMSE of 0.0437. In comparison, when comparing the scaled root weight measurements to the automated segmentation biomass, the RMSE value was equal to 0.0317. With the range of values of the groundtruth variable being 0.2894, we can conclude that in both cases, the predictions are within approximately 0.02 units of the actual values. With the RMSE being significantly smaller than the range of the actual biomass, we can assume that the model is making accurate predictions compared to the variability present in the data. When the algorithm has access to small-size patches, it is able to analyze root structures pixel-wise, which appears to be better equipped for the root segmentation task.

Next, we evaluated the performance of all three RhizoNet models on six root scans, which were “unseen” by the models. Again, we calculated the metrics accuracy, precision, recall, IOU and AUC as in Table 2. Table 1 presents the metrics evaluated on cropped patches, whereas the results in Table 2 correspond to the metrics evaluated on full-size images. In the latter case, the class imbalance highly affects the metric values, and more drastically the IOU.

**Figure 3:**
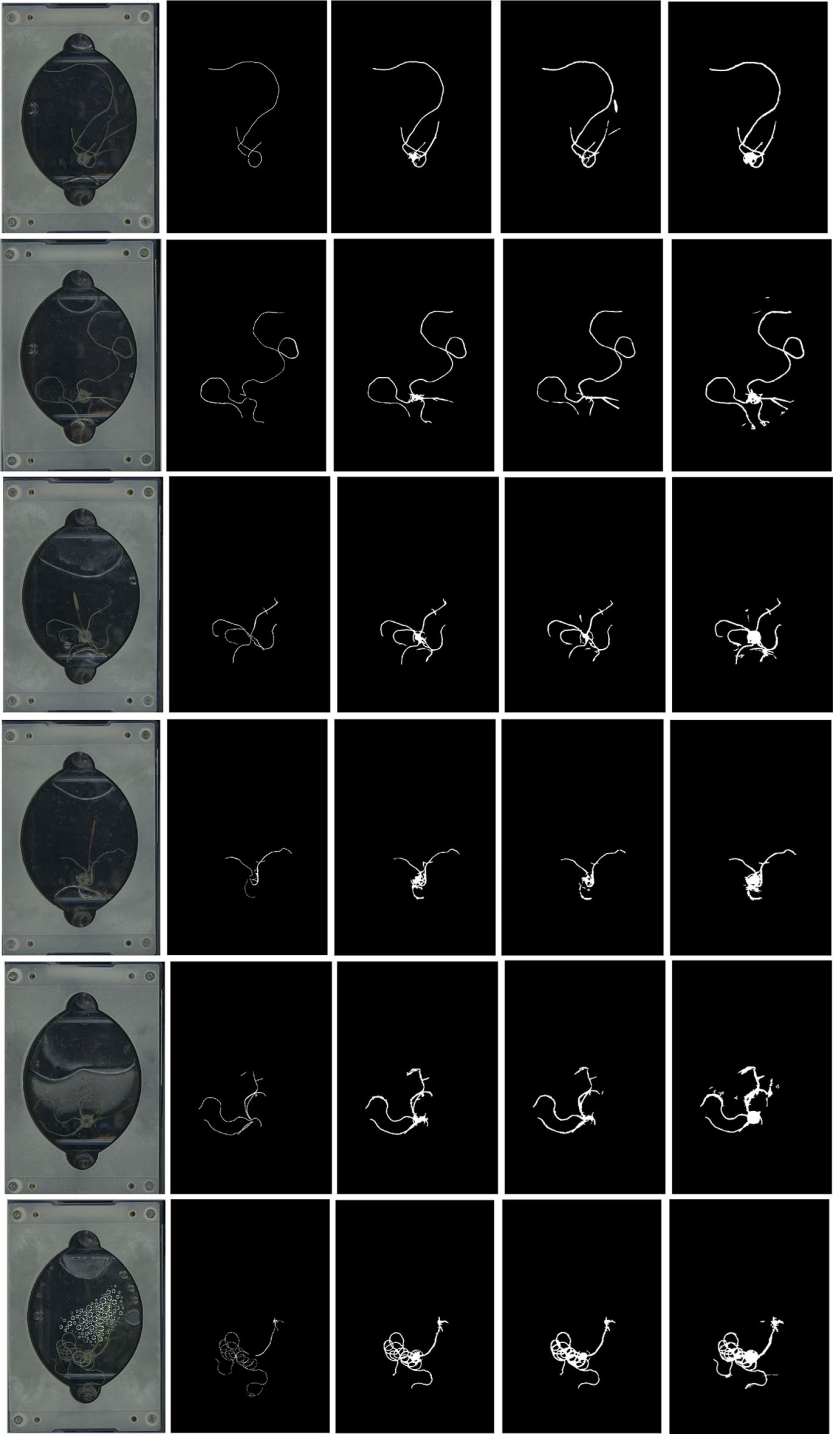
Comparative analysis of 6 unseen images from Exp 2 using three RhizoNet versions. Column 1: Raw images; Column 2: Hand-annotated images; Column 3: Predictions from *Model patch 64*; Column 4: Predictions from *Model patch 128*; Column 5: Predictions from *Model patch 256*.

Furthermore, Fig. 3 and Supplementary Fig. S.1 highlight how well the model performs despite the numerous artifacts surrounding the root, especially for a model trained on patches of size (64,64). Particularly, the superiority of this final model in accuracy over the one trained on larger patch sizes (256,256) is evident when comparing the second and last columns of Supplementary Fig. S.1. The latter demonstrates significantly less noise and artifacts around the root. This model continues to yield the most favorable outcomes, as shown in Table 2. Its training on the smallest (fine) pixel-level features enhances its precision in root segmentation, resulting in a more accurate representation of root thickness compared to the other two models. The model demonstrates high recall, indicating its effectiveness in correctly identifying roots. However, RhizoNet often segments these roots thicker than the ground truth, leading to the misclassification of many adjacent pixels. This discrepancy could stem from root hairs not annotated in the human-generated ground truth or from background pixels near the roots. The precision is relatively low when the scans presented a high level of noise that prevents the model from recognizing the root properly. Additionally, mathematical morphology operations may erode thin parts of the root while removing surrounding artifacts, and the result might also be missing root pixels that contribute to decreasing the recall value.

Based on the observation that RhizoNet tends to overestimate the root thickness, we proceeded to a hyper-parameter search on morphological parameters using the Mean Absolute Error between the thickness of the groundtruth and the prediction; we found that applying additional erosion to the root, using a relatively small structuring element, to decrease its thickness improved the IOU value. We then applied these parameters to the six unseen images and evaluated the metrics of the best model as reported in Table 3. Details of each example are further described in Supplementary Table S.2, using the model trained on patches of size (64,64).

**Table 3:** Evaluation metrics (Accuracy, Precision, Recall, IOU and AUC) of 6 full size unseen images predicted (identical to those used in Fig. 3), processed by each RhizoNet model and in this case, additionally eroded with optimized parameters.

	Accuracy	Recall	Precision	IOU	AUC
Model patch size 64	99.5 ± 0.15	86.03 ± 6.1	43.8 ± 6.6	40.6 ± 5.9	92.7 ± 3.01

## Methods

### Dataset composition and experimental conditions

The dataset consists of images acquired from two separate experiments conducted in various environmental settings. Each experiment featured distinct EcoFAB plants exposed to specific nutritional conditions, including variations in the concentrations of nitrogen, calcium, magnesium, potassium and several micronutrients simultaneously as illustrated in Supplementary Fig. S.2).

The plants under investigation are *Brachypodium distachyon* specimens from a small grass species that serves as a cost-effective research model to study plants. With a small genome size, a short life cycle, and genetic similarity to important cereal crops such as wheat, barley, and oats, it enables the investigation of various aspects of plant biology. Although it is not a major biofuel crop, insights from *B. distachyon* research can inform the development of more efficient biofuel crops by identifying genes and traits related to biomass yield, nutrient utilization, and stress tolerance. Permissions to handle these plants were obtained at Lawrence Berkeley National Laboratory^25^. All methods were carried out in accordance with relevant institutional guidelines, and U.S. national legislation.

The surface sterilized *B. distachyon* seedlings were stratified for 3 days at 4 °C on 0.8% phytoagar plates without nutrients and then germinated in a growth chamber for 3 days before transfer to individual EcoFAB 2.0s (referred herein as simply EcoFABs) that were supplied with growth medium of varying compositions. EcoFABs were assembled as described by Novak et al., For both experiments in which images were acquired, the “Full” control media consisted of 50% strength Murashige and Skoog (MS) growth medium. In both experiments, the pH of the growth media was adjusted to 5.9 (+/−) 0.05 and in Experiment 1 (Exp1), 5 mM MES buffer was added. Changes in the medium were as follows: “N”: ammonium and nitrate free, “P”: phosphate free media, “K”: potassium was removed and supplemented with sodium where appropriate, “Ca”: CaCl2 was removed from the media, “Mg”: MgSO4 was excluded from the medium, “Mic”: all lower concentration nutrients, including Fe-EDTA, boric acid, copper, and manganese, were removed. The “50%” Ca, N and K annotations refer to a reduction in these components to half the concentration of normal 50% media. When not imaged, plants were grown under a 14-h light cycle with photosynthetically active radiation (PAR) levels of approximately 250 (+/− 50) μmols/m^2^/s. Eight images were captured for each EcoFAB plant, covering eight different dates with intervals ranging from 3 to 7 days.

In Experiment 2 (Exp2), which was executed in a setting with a reduced thermostat temperature, two significant observations were made. This environment facilitated better image acquisition of the root system, mainly because the lower temperatures prevented condensation accumulation at the bottom of the EcoFAB plant chamber, thus reducing issues associated with condensation formation. In Exp1, observations were made on all plants over the course of the entire experiment. However, in Exp2, once the plants were determined to be deceased, the imaging was halted. In particular, plants subjected to media without potassium or calcium perished early, so their data sets are shorter (see Supplementary Fig. S.2).

### Image annotation

**Figure 4:**
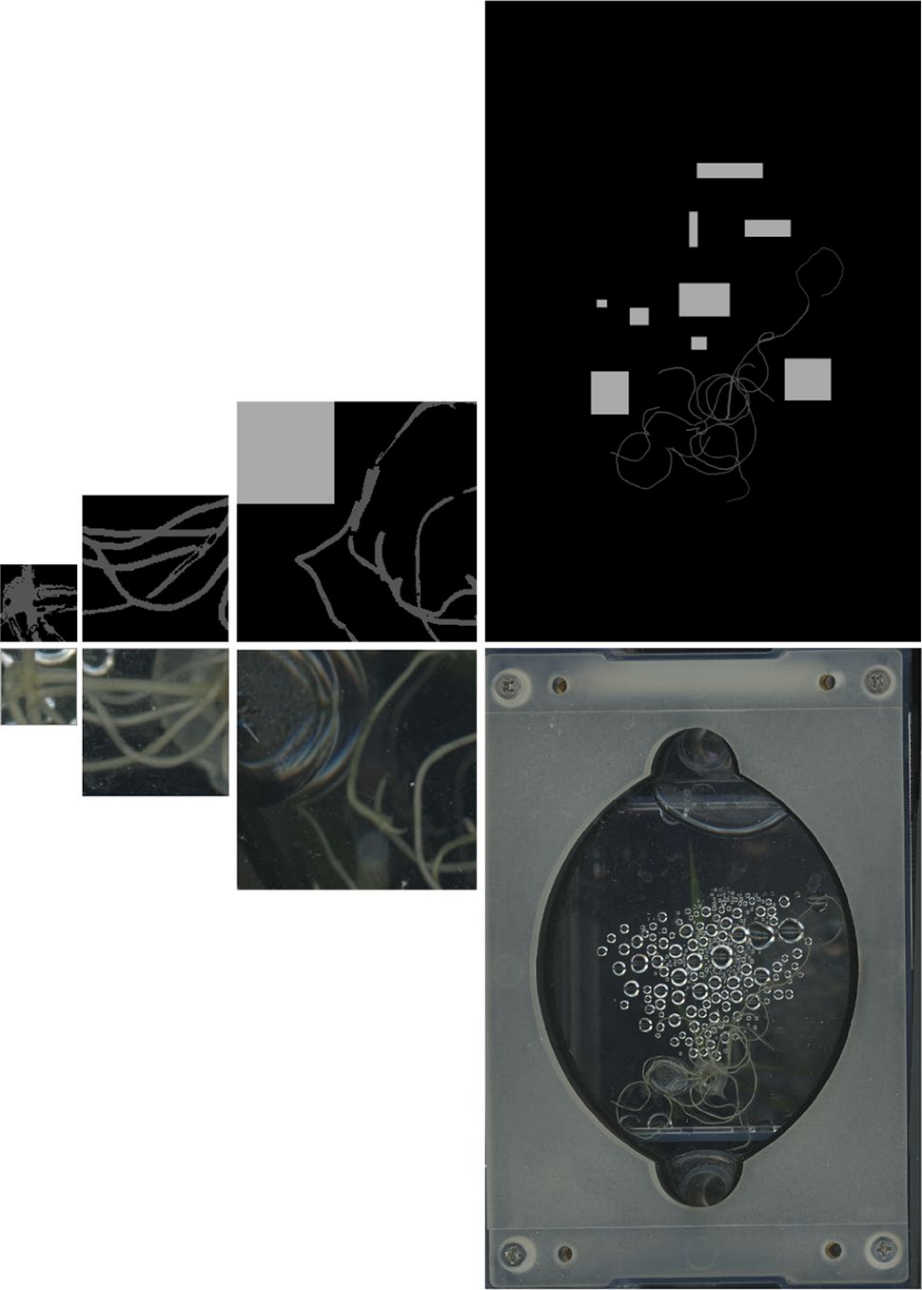
Training patch sample: raw input image at the *bottom* and annotations at the *top*, with varying patch sizes, increasing from the *left* to *right*, corresponding to 64 × 64, 128 × 128, 256 × 256 and full size 3000 × 2039 pixels.

For data preparation, we initiated the process by identifying and labeling three distinct classes: background (class 0), noise (comprising droplets, bubbles, or leaves, labeled class 1) and root (class 2), being one example of a labeled image found in Fig. 4. Unlabeled data were utilized to make unbiased predictions leveraging the weights from the trained model, so that one can assess its effectiveness on data that it had not encountered before.

The ground-truth biomass measurements were conducted using a scale (balance) to measure the root weights for both experiments to the nearest milligram (mg). However, since these are destructive measurements, they were performed exclusively on the last acquisition date for each EcoFAB. Consequently, the ground truth biomass data only correspond to the final EcoFAB imaging dataset.

Analogously, the biomass of manually annotated images was determined by quantifying the number of pixels corresponding to the root. Following this, the obtained value was normalized using the default L2 norm parameter of the normalization function from the *sklearn* library, applied across all EcoFAB images captured on the latest acquisition date. This normalization process was also applied to the ground truth biomass values, enabling comparative and analytical evaluations. It is important to note that there may be some degree of uncertainty associated with individual root annotations, which should be taken into account when interpreting the results further.

Our analysis commenced with an evaluation of the biomass of the manual annotations concerning the ground-truth biomass (balance) of the final acquisition date within the second dataset. We performed a regression analysis, initially fitting a model between the labeled images and the ground-truth. This approach provided correlation values, including specific correlations to different nutritional deficiencies (Supplementary Table S.3), further discussed in the “Results” section (refer to Fig. 2).

### Data analysis

The images annotated by the human experts compose the training dataset, consisting of 61 original scans of size (3000, 2039) extracted from the sixth and eighth acquisition dates of Exp 2, and 61 grayscale annotated target images (see Fig. 4 last column). Given that the raw data contain noise and include irrelevant parts due to condensation, bubbles, and shadows, we proceed to develop an algorithm that includes routines to ignore those regions.

Therefore, to enable the algorithm to focus on the lower-level features of the root and better understand its structure, we created three different types of patches of sizes (64,64), (128,128) and (256,256). We considered 2D models capable of handling RGB images and subdivided each training hand-labeled slice and raw image into $128\times 128$ patches (refer to Fig. 4), $64\times 64$ patches and $256\times 256$ patches, which were then separated into 80% training, 10% validation, and 10% testing datasets. This processing was especially helpful given that each image was highly imbalanced pixel-wise since the very thin roots occupied a very small part of the (3000, 2039) raw images.

In addition to creating a training dataset of patches, we also executed data augmentation schemes in training all models, consisting of random rotations in all directions, random flips (vertically and horizontally), random cropping (2%), random shifts, random zoom within range [0.8, 1], and a small range of random brightness and contrast variation (± 5%). These geometric transformations are applied at run-time before the training of the RhizoNet begins. This data augmentation aims at diversifying the training dataset and reducing overfitting in the model.

### Computational models

Our software tool leverages PyTorch, a leading open-source machine learning library, as a foundational framework. Building upon this, we integrate MONAI (Medical Open Network for AI), which is an advanced open-source framework based on PyTorch and tailored specifically for healthcare imaging. MONAI provides specialized functionalities essential for developing deep learning techniques^26,27^, including data loaders and routines designed to enhance the efficiency of code processing. Complementing these capabilities, our tool also builds upon the scientific Python stack, incorporating key libraries such as scikit-learn for machine learning and matplotlib and seaborn for time-series visualization. Essentially, the approach leverages GPU-accelerated algorithms to enhance the processing speed as it handles multiple crops in parallel. This enhancement allows RhizoNet to rapidly process large image data sets, aligning with the rigorous requirements of plant scientific research. The creation of the RhizoNet model benefits from high-performance resources at NERSC, by using a node of the system called Perlmutter. It enables accelerated training using the NVIDIA Tesla V100 GPU for each experiment, the model size was roughly 26 MB for 6.5 million parameters. The following Table 4 summarizes the number of patch samples for each training and the associated duration of training.

**Table 4:** Patch sizes, number of patches used during training and training time for creation of RhizoNet models—sample size before augmentation.

Patch size	Sample size	Training time
(64,64)	38,176	30 min
(128,128)	10,943	45 min
(256,256)	3209	1 h

### Deep learning: residual U-Net

The U-Net is a convolutional neural network with an architecture that was first introduced in 2015 by Ronneberger et al. for the semantic segmentation of biomedical images. The original work^17^ proposes an architecture that consists of a contracting path to capture context and a symmetric expanding path that enables precise localization. In our work, the model was adapted to take images of different sizes as input (sizes available in Table 4). For the encoder, we started with 32-channel with $3\times 3$ kernels but each standard block was replaced by a 2-convolution residual unit, which is detailed in the next section.

**Figure 5:**
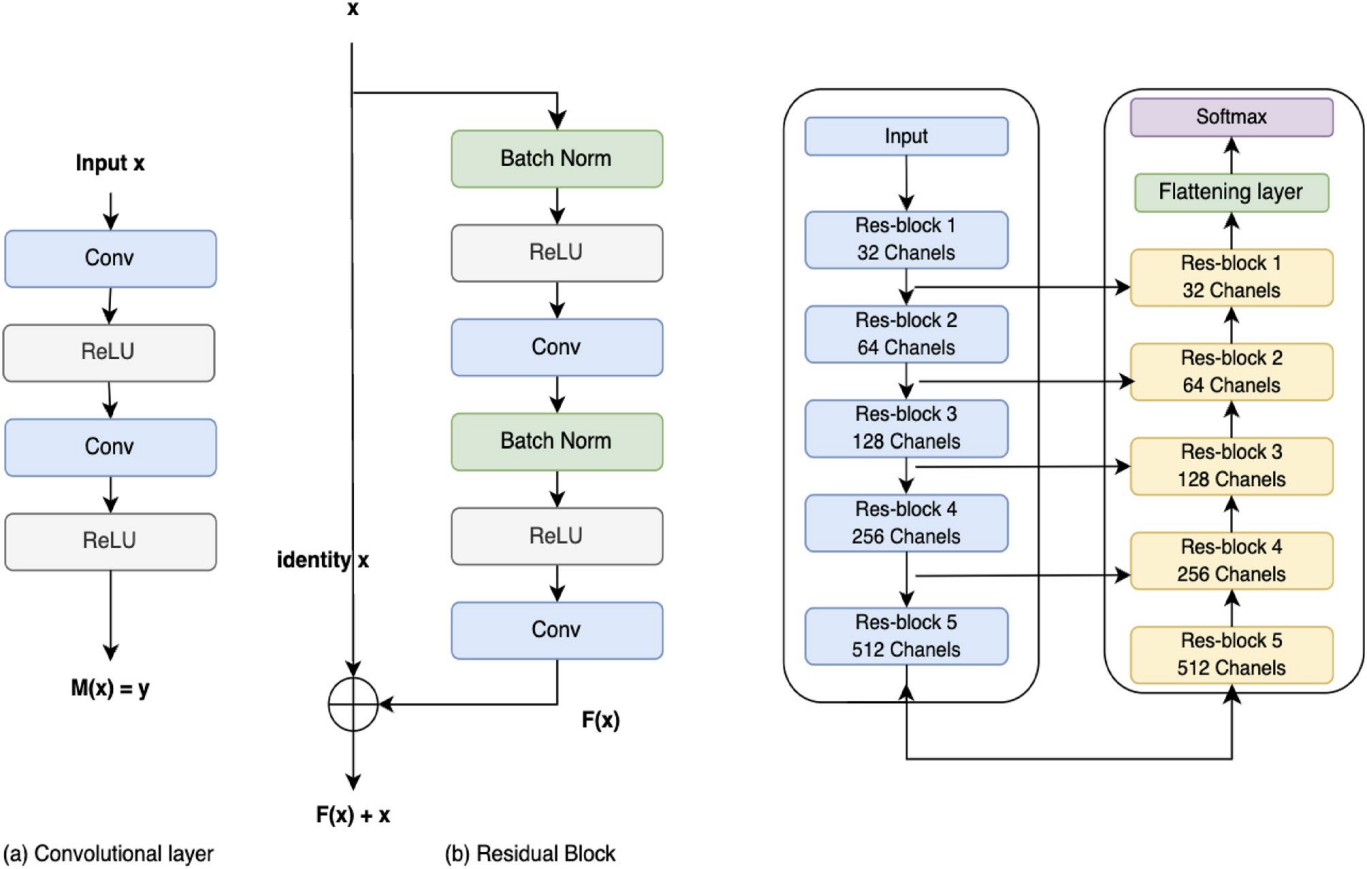
Building blocks of the Residual U-Net architecture with (left image) in (**a**), plain neural unit used in a standard U-Net and (**b**), a residual unit with identity mapping and (right image) the full U-Net architecture with Encoder–Decoder components.

An enhanced version of U-Net is the Deep Residual U-Net, which consists of two main components: the U-Net architecture itself with an encoder that downsamples the image features at multiple scales and a decoder that upsamples these features to produce a segmentation map with the same resolution as the input. Each block of the encoder and the decoder is connected by skip connections, allowing the decoder to access information from different scales. Both the encoder and decoder consist of a 5 layer network with, respectively, downsampling and upsampling by a factor of 2 at each layer. The Residual U-Net combines the concepts of residual networks (ResNets) and the U-Net architecture such that each layer in the encoder and decoder is implemented as a residual block (Fig. 5) which contains skip connections that allow the network to learn residual information: each block consists of a residual unit with 2 subunits. Each subunit has a batch normalization, rectified linear unit (ReLU) activation function, and convolution layers with 3 × 3 kernel filters for each convolution. An additional 20% dropout was applied.

The output of the decoder is a logit vector, we then use the Softmax function given that we are classifying into 3 separate classes and thus the loss function used is the weighted cross-entropy loss. Indeed, as we mentioned previously, the data set is highly imbalanced, which is why it was necessary to add the class weights in the loss function. The evaluation metrics considered here are the precision, recall, accuracy, and IOU (Intersection Over Union) coefficient.

The Softmax function, cross-entropy and balanced cross-entropy losses (Eq. (1)) are defined as follows:

1 $$\begin{aligned} \text {Softmax}(z_i)&= \frac{e^{z_{i}}}{\sum _{j=1}^K e^{z_{j}}} \ \ \text {for}\ i=1,2,\dots ,K \nonumber \\ \mathscr {L}_{\text {Cross Entropy}}(y,\hat{y})&= -\sum _{c=1}^My_{o,c}\log (p_{o,c}) = -\sum _{c=1}^My_{o,c}\log (\hat{y})\nonumber \\ \mathscr {L}_{\text {balanced Cross Entropy}} (y,\hat{y})&= -\sum _{c=1}^M\beta y_{o,c}\log (\hat{y}) \end{aligned}$$

where M is the number of classes (in this case 3), log is the natural log, y is the binary indicator (0 or 1) if class label c is the correct classification for observation o, and p is the predicted probability that observation o is in class c (we can also write $\hat{y}{o,c} = p_{o,c}$. In balanced cross-entropy, $\beta$ represents the weight of each class and where $\beta \in [0, 1]$. We also define the IOU as in Eq. (2) below:

2 $$\begin{aligned} \mathscr {I}\text {o}\mathscr {U}(y,\hat{y}) = \frac{TP}{TP + FP + FN} \end{aligned}$$

### Data post-processing and final predictions

The proposed algorithm improves segmentation results by also exploring the EcoFAB images to define the key region-of-interest (ROI). It combines prior knowledge about plant root evolution in these specialized hydroponic systems. To minimize the noise surrounding the segmented root, we employed mathematical morphology operations^28,29^, starting with dilation on the stacked segmented root image obtained from predictions on all EcoFAB acquisition dates. We then extracted a convex hull from the segmented roots and applied area closing and area opening transformations.

To calculate the convex hull of a set of points, we used the Graham’s Scan algorithm, which main steps are as follows:

- Identify a pivot point, usually the point with the lowest y-coordinate (and leftmost if tied).
- Sort all points by their polar angles with respect to the pivot.
- Initialize an empty stack to store convex hull points.
- Iterate through the sorted points and add them to the stack while ensuring a counterclockwise order.
- The stack’s content represents the convex hull vertices.

We used Python libraries such as ‘scipy‘ to efficiently compute the convex hull, which finds the smallest convex polygon enclosing all the given points.

Utilizing a convex hull for ROI delimitation consists of employing a geometric shape that encompasses a set of points while forming a convex polygon with the minimum possible area. In this context, the points represent the set of stacked roots (maximum intensity projection of timeseries prediction) for a specific EcoFAB, determined by the coordinates of the root pixels. The convex hull algorithm determines the outermost boundary that encloses all the points, forming a convex shape. By comparing the convex hull with the original stacked noisy image, we were able to identify and remove noise located outside the hull. Through the combination of the convex hull method and morphological operations, we effectively eliminated the noise surrounding the root at each time point (Supplementary Fig. S.3 bottom right image).

Finally, Supplementary Fig. S.4 points out the difficulty in segmenting the root and evaluating the most accurate thickness, more precisely in the case of highly noisy images. It seems that some images are too complex for the algorithm to segment and even more complex for the human eye, since we can hardly differentiate and identify droplets, very light-colored leaves, and other artifacts with roots. Therefore, the final step consisted of optimizing the post-processing morphological parameters to obtain the most accurate root thickness and compare once again the final results with the groundtruth (see results in Table 3 in “Results” section).

## Discussion

Our primary findings underscore the different challenges encountered when working with EcoFAB images, including issues related to condensation, bubbles, reflections, leaf shadows, and root overlaps. These intricacies make conventional pixel-based methods, such as thresholding and random forests, inadequate for the task. Therefore, a more robust and customized solution becomes imperative. Our study highlights the adaptability of the RhizoNet model, demonstrating its ability to effectively handle morphologies resulting from root growth over time and to discern and separate root plants from unwanted noise in most cases, resulting in more precise and accurate root segmentation.

In a comparative analysis with manual annotations, RhizoNet demonstrated superior performance, particularly in IOU and accuracy metrics. By constructing features that focus on low-scale, fine details, and nuances within each small patch, RhizoNet enhances detail capture and local feature learning, resulting in a more robust and reliable model. This advanced capability of RhizoNet to effectively generalize paves the way for enabling self-driving labs.

Furthermore, the results of the two experiments underscore the critical role of environmental parameters during plant growth, especially in relation to the specific concentration of nutrients. This suggests that refinement of these parameters could affect the results of the project. Additionally, exploring smaller patch sizes is a promising avenue, since the best results were achieved with a patch size of 64, the smallest among the three tested sizes. Smaller sizes may offer improved feature encoding capabilities within the model.

We also find that the Residual U-Net allied to convex hull operators augments our ability to assess root biomass and plays an essential role in the segmentation pipeline: the use of convex hull improved the model’s ability to focus on the best ROI, helped to eliminate noisy artifacts and improved root segmentation. This breakthrough has far-reaching implications, as it not only enables accurate root biomass calculations, but also highlights the potential for transferring knowledge and models across domains.

**Figure 6:**
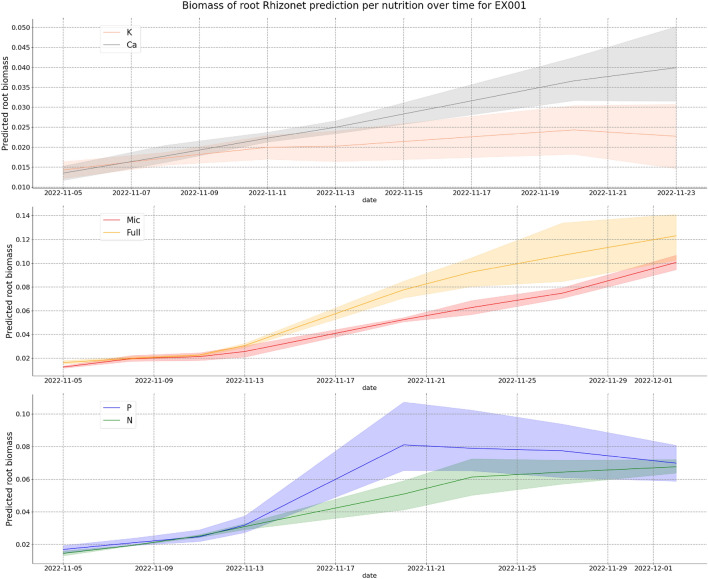
Root growth plot per nutrition deficiency for Exp1.

**Figure 7:**
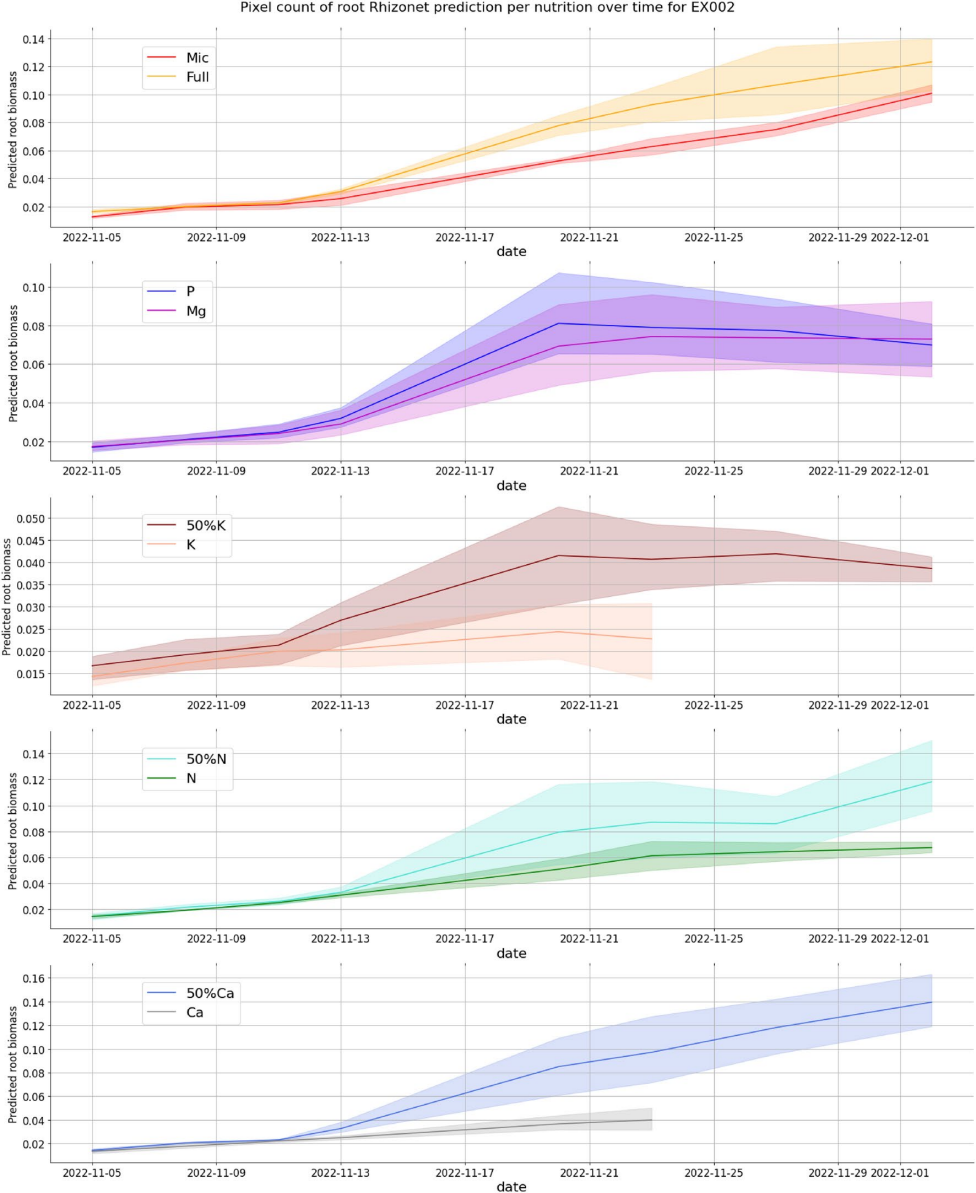
Root growth plot per nutrition deficiency for Exp2.

The unlabeled data served a crucial role in extending the utility of our model. By utilizing these data to make additional predictions with the weights of the trained model, we were able to visually assess its performance on the data not used during training (see Fig. 3 and Supplementary Fig. S.1). This evaluation not only demonstrated the model’s capacity for generalization, but also showcased its potential for the learned model to be applied to multiple experiments. We were then able to obtain information on root growth and plant responses for both Exp1 and Exp2 (see Figs. 6 and 7). In particular, two specific plants with potassium and calcium deficiencies died before the last date in Exp 2, probably due to lower temperature settings in the growth room. In contrast, most of the 40 EcoFABs in Exp 1 reached their last date, and the majority exhibited positive growth. This highlights the critical influence of environmental factors, including temperature and nutrient availability, during plant growth. We also observe with Exp2 (Fig. 7) how the decrease in essential nutrient such as Calcium or Potassium negatively impacts root growth over time.

### Supplementary Information

Supplementary Information.

## Data Availability

Python codes for root scans segmentation enabled by RhizoNet were created by the authors and are described in this paper. These codes will be available free of charge upon acceptance, and with open source at: https://github.com/lbl-camera/rhizonet.
