# Supplementary material for: RhizoNet segments plant roots to assess biomass and growth for enabling self-driving labs
Source: Sci Rep. 2024 Jun 5;14:12907. doi: 10.1038/s41598-024-63497-8 (PMC11153571; doi:10.1038/s41598-024-63497-8)
Supplement: Supplementary file 1 — Supplementary Information. [file 41598_2024_63497_MOESM1_ESM.pdf]

Supplemental Material

RhizoNet Segments Plant Roots to Assess Biomass and Growth for Enabling Self-Driving Labs

A Comparative Analysis of RhizoNet Results

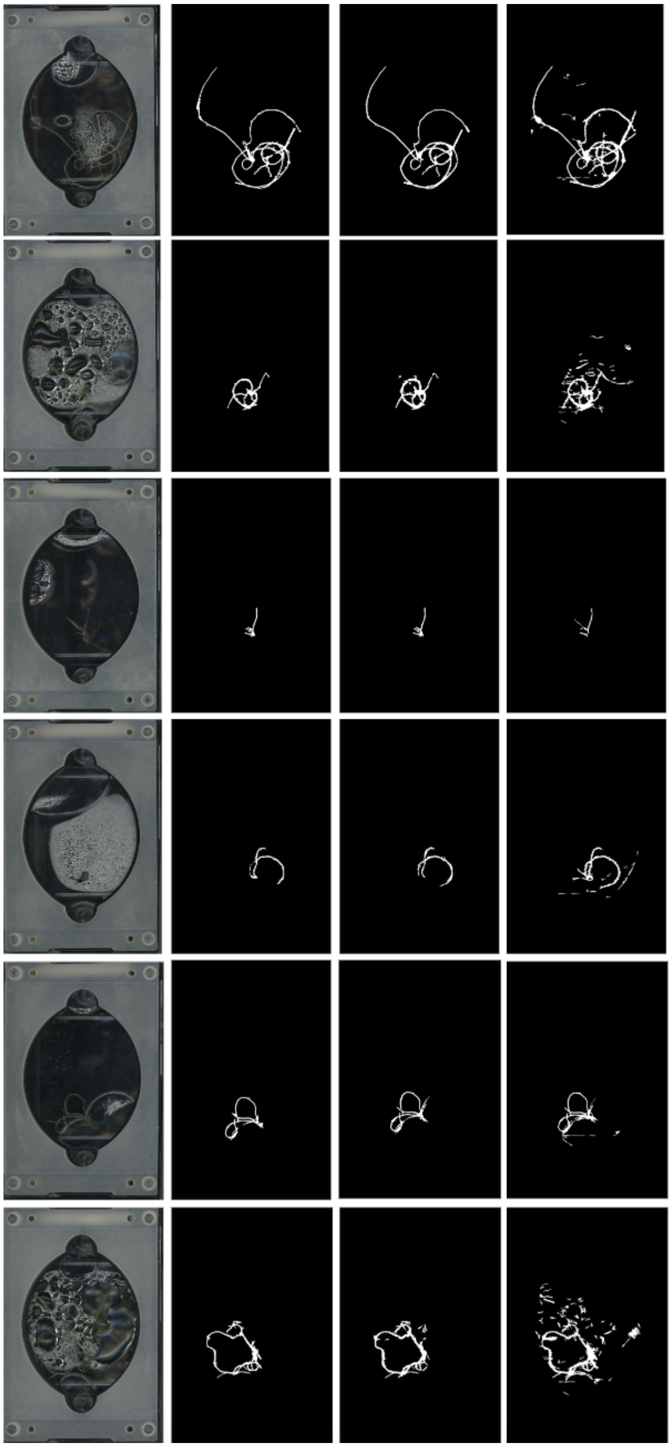

Figure S.1. Comparative figure of the predictions of 6 images of `Exp 1` unseen by the model using 3 versions of RhizoNet: columns 1 is the raw images, column 2 is the processed prediction of *Model patch 64*, column 3 is the processed prediction of *Model patch 128*, column 4 is the processed prediction of *Model patch 256*.

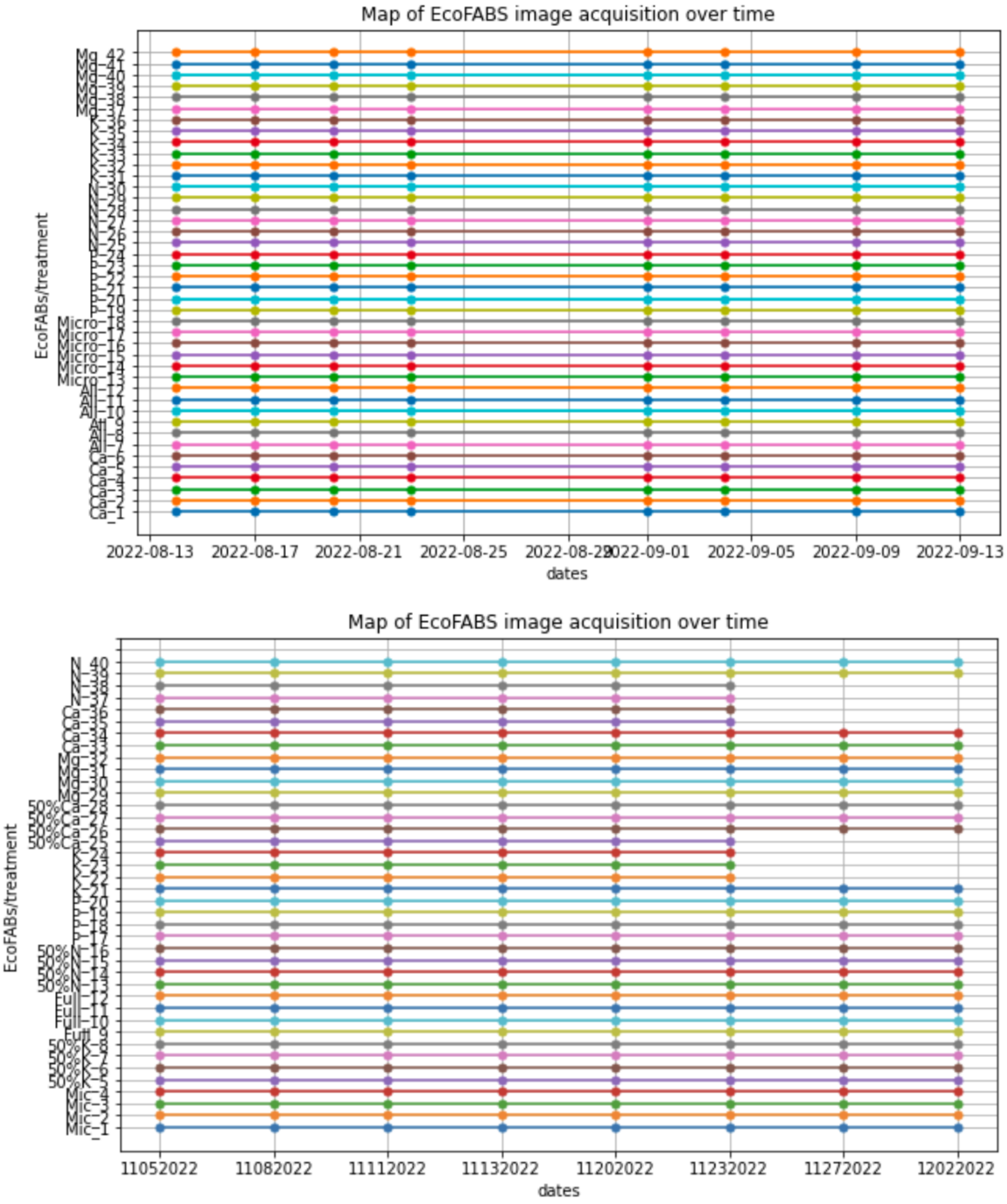

Figure S.2. Timeseries of images in `Exp 1` (top) and 2 (bottom), representing each EcoFAB scan as a circle. This plot illustrates each data point used for training and testing, obtained from two distinct experiments conducted in various environment settings where each EcoFAB plant was exposed to specific nutritional conditions, including variations in the concentrations of nitrogen, calcium, magnesium, potassium and several micronutrients simultaneously. In particular, `Exp 2` led to the early death of 2 specific plants: plants grown in media without potassium or calcium perished early, so their data sets are shorter (bottom figure).

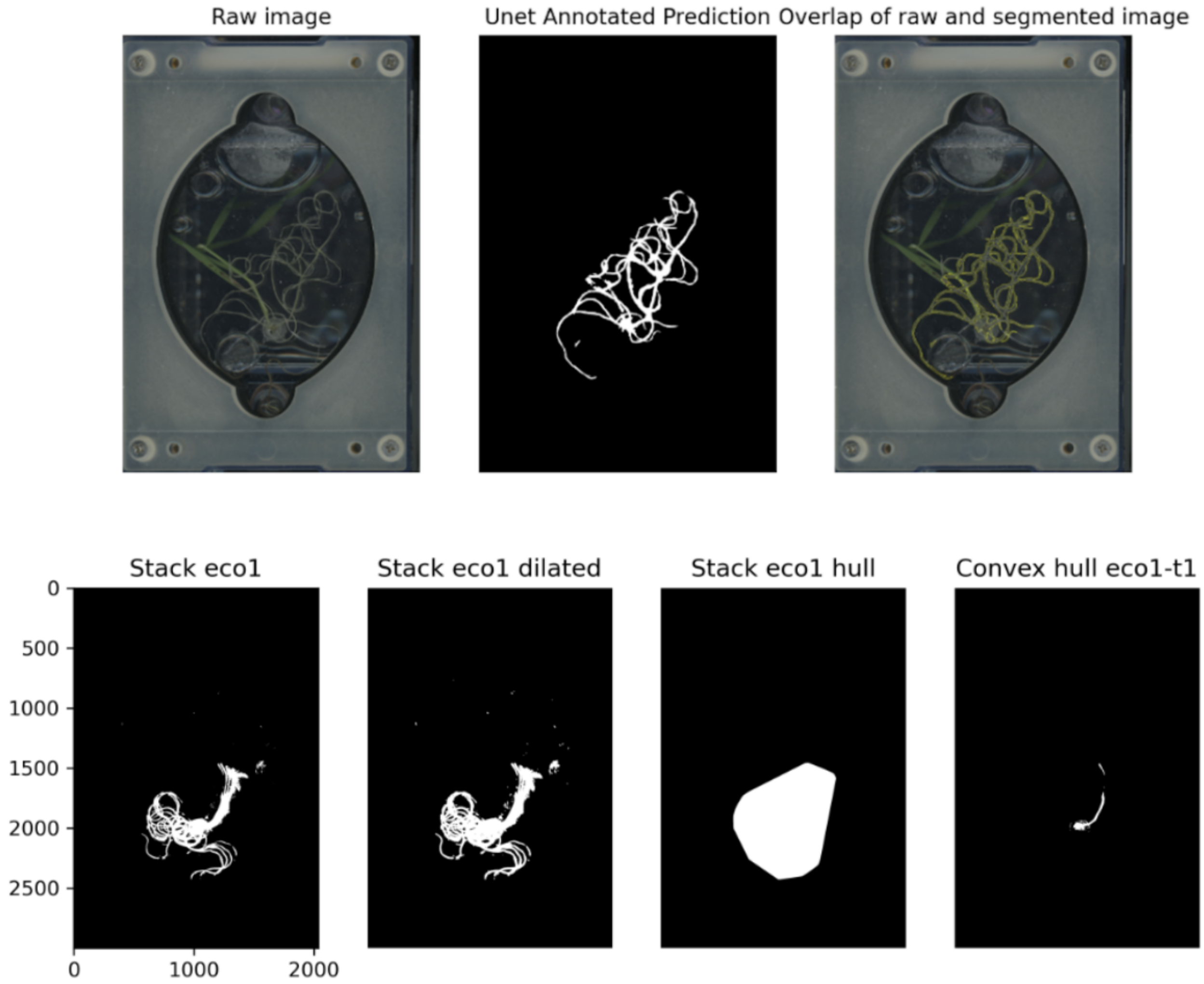

Figure S.3. Top: raw, prediction and overlap of segmentation with raw image. Bottom: stack of all prediction masks for a given EcoFAB that is the images at each date for each EcoFAB plant, morphological dilation applied to the stacked result, convex hull created using the stacked result and final prediction after removal of all surrounding noise using the previously generated convex hull. This post-processing method explores all future positions of the root for a given EcoFAB and thus optimizes the noise removal process.

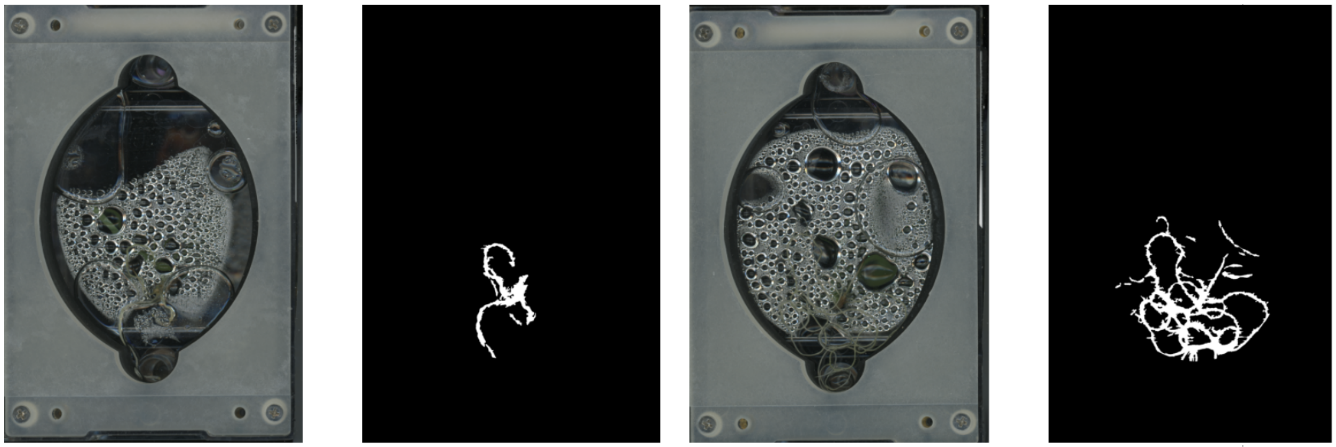

Figure S.4. Images with the thickest segmented roots and their associated raw image. Root thickness is slightly overestimated when the image is particularly noisy with condensation and other artifacts very similar to the root structure and color.

		Accuracy	Precision	Recall	IOU	AUC
	**1**	99.8	90.8	24.8	24.19	94.8
	**2**	99.2	99.3	32.3	32.3	99.6
	**3**	99.5	96.9	33.5	33.2	98.2
**Model Patch size 64**	**4**	99.4	98.2	40.4	40.1	98.9
	**5**	99.3	96.1	43	42.3	97.7
	**6**	99.3	99.6	31.1	31	99.4
	**Average**	**99.25 $\pm$ 0.24**	**96.82 $\pm$ 3.24**	**34.18 $\pm$ 6.6**	**33.85 $\pm$ 6.55**	**98.1 $\pm$ 1.77**
	**1**	98.5	90.7	21	20.6	94.6
	**2**	99.2	99.1	32.3	32.2	99.2
	**3**	99.6	93.3	37.7	36.7	96.4
**Model Patch size 128**	**4**	99.4	94.1	37.4	36.5	96.8
	**5**	99.1	91.5	38.5	37.2	95.3
	**6**	99.1	99.95	25.7	25.7	99.5
	**Average**	**99.15 $\pm$ 0.37**	**94.78 $\pm$ 3.88**	**32.1 $\pm$ 7.27**	**31.48 $\pm$ 6.89**	**96.97 $\pm$ 2.01**
	**1**	98.1	94.8	17.7	17.5	95.5
	**2**	98.5	99.9	20	20	99.2
	**3**	99.2	99.9	22	22	99.5
**Model Patch size 256**	**4**	98.8	99.7	24.4	24.5	99.2
	**5**	98.5	99.9	26.3	26.3	99.2
	**6**	98.8	100	21.2	21.1	99.4
	**Average**	**98.65 $\pm$ 0.37**	**99.03 $\pm$ 2.08**	**21.93 $\pm$ 3.08**	**21.9 $\pm$ 3.15**	**98.67 $\pm$ 1.56**

Table S.1. Evaluation metrics (Accuracy, Precision, Recall, IOU and AUC) of 6 full size unseen images of `Exp 2` predicted and processed by each RhizoNet model. This table details results of Table 2 for each image.

B Data Availability

B.1 Grass as a research model

The roots of *B. distachyon* plants were subjected to various conditions of deprivation of nutrients for approximately 5 weeks. Figure S.2 summarizes the time points where images were captured. Each line represents a plant growing in its respective EcoFAB chamber, which is monitored for days that are represented by solid circles.

To capture images of plant roots, the lower section of each EcoFAB undergoes a scanning process, as depicted in Fig. S.5 and detailed in the next section.

		Accuracy	Recall	Precision	IOU	AUC
**Model Patch size 64**	**1**	99.2	88.1	33.2	31.8	93.6
	**2**	99.5	96.7	42.4	41.8	98.1
	**3**	99.7	79.3	38.5	34.9	89.5
	**4**	99.4	84.5	52.4	47.8	92.1
	**5**	99.5	78.9	54.03	47.2	89.3
	**6**	99.6	88.6	42.3	40.2	94.1
	**Average**	**99.5 ± 0.15**	**86.03 ± 6.1**	**43.8 ± 6.6**	**40.6 ± 5.9**	**92.7 ± 3.01**

Table S.2. Evaluation metrics (Accuracy, Precision, Recall, IOU and AUC) of 6 unseen images predicted, processed by each RhizoNet model and additionally eroded with optimized parameters. This table details results of Table 3 for each image.

	Nutrition Deficiency	Pearson correlation
1	50%K	-0.8797
2	50%N	-0.506
3	Mg	-0.3870
4	50%Ca	0.0236
5	P	0.6057
6	K	0.7008
7	Full	0.7180
8	Micronutrients	0.8924
9	N	0.9099
10	Ca	0.9858

Table S.3. Correlation values for each nutrition condition observed in `Exp 2` with sufficient replication in the final (12/02/2022) dataset. Correlations were performed between the normalized pixel counts, manually identified, and the mass measured for the corresponding plants

B.2 Data acquisition and inherent artifacts

For image acquisition, we used the EPSON Perfection V850 Pro. This professional-grade scanner is designed for advanced scanning applications, offering exceptional precision and quality when scanning paper sheets and various other media types. The key features of the EPSON Perfection V850 Pro include a versatile scanning solution with its dual high-resolution lens system, capable of scanning photos, documents, and 35mm film and slides with optical resolution of up to 6400 dpi, its wide compatibility with both Windows and Mac operating systems makes it accessible to a diverse range of users.

Despite our ability to achieve high-quality scans, the images of plant roots generated during the EcoFAB-based plant growth process present intricate challenges for root detection. This process introduces various issues, including condensation, bubbles, and shadows illustrated in Fig. S.5). Condensation in plant imaging arises from the formation of water droplets on surfaces of the EcoFAB chamber due to plant transpiration that can then accumulate due to temperature disparities between the equipment and its environment. This frequently occurs in environments with temperature variations, such as growth chambers, where warm, humid air contacts cooler surfaces. The condensation issue becomes particularly problematic during extended imaging sessions, notably in the later frames of the time series (refer to Fig. S.5). Similarly, but on a larger scale, bubbles can form due to plant roots, primarily through oxygen release, which benefits the microorganisms in the root zone but can obstruct plant root visibility in the proposed imaging system.

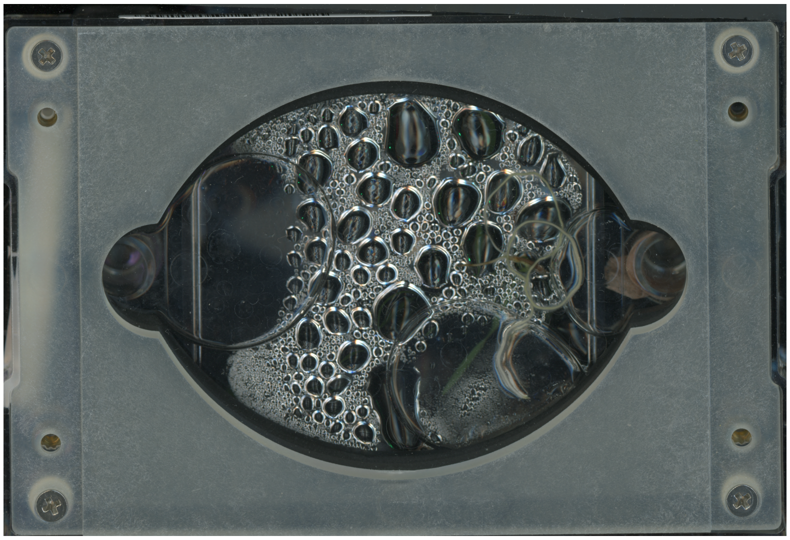

Figure S.5. Non-processed EcoFAB image after two weeks: acquired on September 1st 2022, with visible condensation and bubbles.

These physical phenomena pose difficulties for segmentation algorithms, which may misinterpret subtle droplet edges as roots. Furthermore, leaf shadows also introduce background noise, complicating the distinction between thin leaves and the target for both algorithms and human observers due to their pixel-wise resemblance to roots.

To mitigate these artifacts, two experiments were carried out, namely `Exp 1` and `Exp 2`, using a *B. distachyon* seedling per EcoFAB and dozens of EcoFABs per experiment, as described in Table S.4.

Exp	Nb of EcoFABs	Nb of scans	Nb of training scans	Nb of val/test scans	Nb of unseen scans
1	40	337	N/A	N/A	337
2	42	306	61	10	245

Table S.4. Distribution of plant root scans in EcoFABs acquired during different experiments: Exp = Experiment number, Nb = number

B.3 Comparison with RootPainter:

Using a subset of our training data, we used RootPainter for root segmentation and we followed the suggested steps provided by the software tutorial. In order to enable a fair comparison, we took additional preprocessing steps necessary to meet the input type, size and shape requirements for the utilization of the software. Given that our dataset already had a set of annotations, we first trained RootPainter using these annotations without additional corrective labeling which amounts to training a U-Net architecture with the software default train and test settings. The steps are the following:

- Convert our annotations, which carries information about 3-classes in 3 different shades of gray (Fig. S.6 *left* image), into binary masks containing only black and blues pixels (Fig. S.6 *middle* image), being black for background and blue for foreground, as suggested by the RootPainter GitHub discussion (https://github.com/Abe404/root_painter/discussions/108). This step was necessary before being able to use the converting tool of RootPainter that enabled the creation of red and green annotations (Fig. S.6 *right* image), which is the standard annotation form.

- Use the software function *convert segmentation to annotations* to obtain the following result:

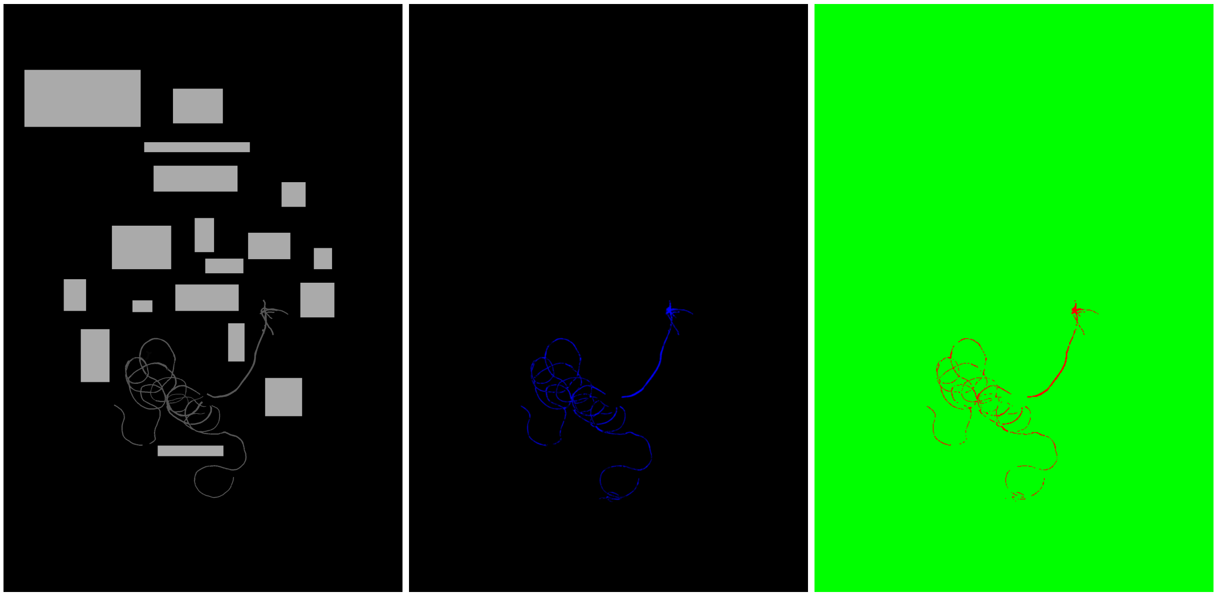

Figure S.6. Converted annotations: *left* is the original annotation, *middle* is the binary image with blue root and black background and *right* is the RootPainter adapted binary annotation with red root and green background.

- Resize the image from *3000x2049* to *600x600*: instead of resizing, RhizoNet creates smaller size patches prior to training which maintains the high image resolution and improves model performance.
- Run the software with 21 annotations used in the train set and 10 in the validation set, which lead to the following results (without corrective annotation) given in Fig. S.7:

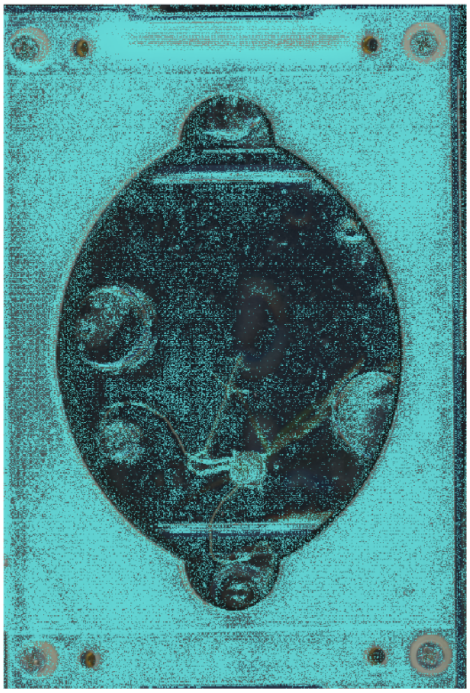

Figure S.7. Segmentation result with 21 training images and without corrective labeling.

- Finally, instead of using the available annotations, we trained the model by using the corrective labeling tool from scratch as suggested in the tutorial and after 17 manual segmented images used for training (the suggested minimum number

by RootPainter being 7 images). We obtained a low F1 and precision score and a high recall after 34 out of maximum allowed 60 epochs. Next, we iteratively refined the RootPainter results for 18 cycles, obtaining Fig. S.8 *left*, which we compared with the results of RhizoNet when only performing preprocessing, as shown in Fig. S.8 *middle* and when both preprocessing and postprocessing were considered as in Fig. S.8 *right*.

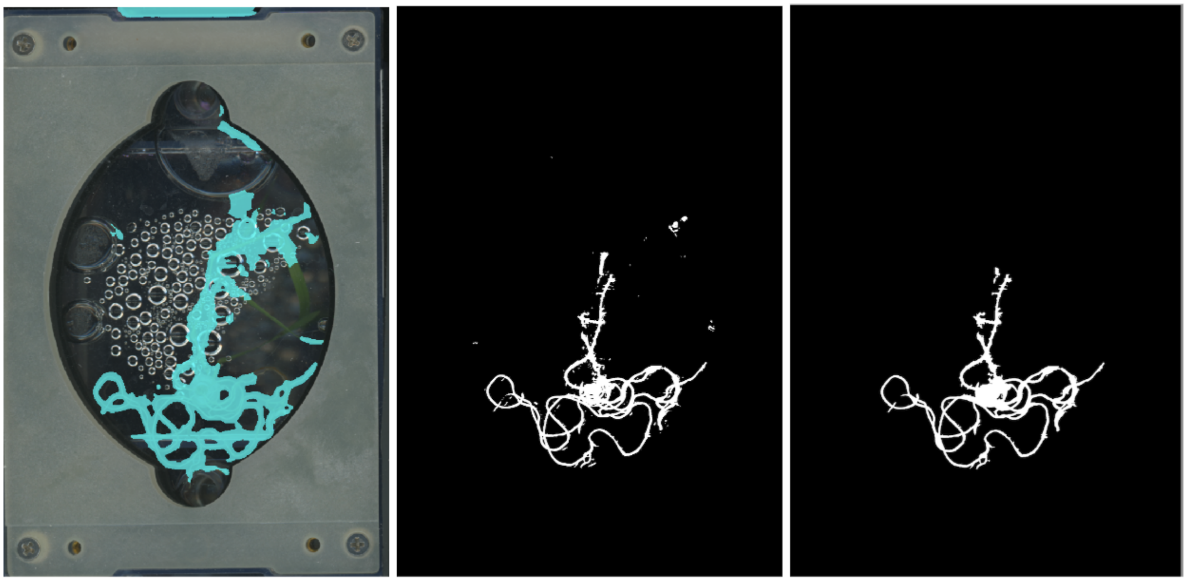

Figure S.8. Comparison between the 18th Rootpainter segmentation (*left*), preprocessed (*middle*) and postprocessed (*right*) predictions using RhizoNet.

In summary, the use of RootPainter for EcoFAB plant root segmentation seems to be suboptimal for the purpose of self-driving labs as well as the use of high performance computing. Other issues encountered are:

1. **Preprocessing Enhancements**: Our experiments revealed that preprocessing steps play a crucial role in the performance of our model. For RhizoNet, we utilized smaller size patches and MONAI-based data augmentations (such as rotation, dilation, and flipping), which significantly improved the handling of variations in input data. This was not possible with RootPainter as it does not support the use of patches for training and instead requires training on full-size images, which resulted in suboptimal segmentation results for complex root structures.

2. **Limitations with RootPainter's Preprocessing**: RootPainter's inability to handle large-size images required us to resize our high-resolution images (3000x2049), leading to a loss of detail and further compounding the segmentation challenges. The corrective training feature in RootPainter, although interactive, did not sufficiently compensate for the initial poor quality of segmentation, even after multiple corrective annotations.

3. **Postprocessing Techniques**: The postprocessing steps we developed for RhizoNet, particularly the use of a convex hull method over time-series data to refine segmentation and remove noise, significantly enhanced the clarity and accuracy of our results. While theoretically, similar postprocessing could be applied to RootPainter, the preliminary segmentation quality was not adequate to benefit fully from these advanced techniques. In practice, a major modification to RootPainter software would need to take place, which is outside of the scope of this paper.

4. **Comparative Results**: We included comparative imagery that visually demonstrates the superior segmentation performance of RhizoNet over RootPainter. The images show the segmentation results from RootPainter after 17 corrective annotations versus the preprocessed and postprocessed results using RhizoNet, clearly illustrating the enhancements brought about by our method.

5. **Overall Performance**: In summary, the architectural differences in RhizoNet, particularly the use of a Residual UNet, combined with our specialized preprocessing and postprocessing steps, allow it to outperform RootPainter under similar conditions. RootPainter's model cannot be enhanced to the same degree within its current framework, indicating that RhizoNet offers intrinsic benefits that lead to superior performance when dealing with images from EcoFABs acquired by the EcoBOT.
